# Differential network analysis from cross-platform gene expression data

**DOI:** 10.1038/srep34112

**Published:** 2016-09-28

**Authors:** Xiao-Fei Zhang, Le Ou-Yang, Xing-Ming Zhao, Hong Yan

**Affiliations:** 1 School of Mathematics and Statistics & Hubei Key Laboratory of Mathematical Sciences, Central China Normal University, Wuhan, 430079, China; 2 Department of Electronic Engineering, City University of Hong Kong, Hong Kong, China; 3 College of Information Engineering, Shenzhen University, Shenzhen, 518060, China; 4 Department of Computer Science, School of Electronics and Information Engineering, Tongji University, Shanghai, 201804, China

## Abstract

Understanding how the structure of gene dependency network changes between two patient-specific groups is an important task for genomic research. Although many computational approaches have been proposed to undertake this task, most of them estimate correlation networks from group-specific gene expression data independently without considering the common structure shared between different groups. In addition, with the development of high-throughput technologies, we can collect gene expression profiles of same patients from multiple platforms. Therefore, inferring differential networks by considering cross-platform gene expression profiles will improve the reliability of network inference. We introduce a two dimensional joint graphical lasso (TDJGL) model to simultaneously estimate group-specific gene dependency networks from gene expression profiles collected from different platforms and infer differential networks. TDJGL can borrow strength across different patient groups and data platforms to improve the accuracy of estimated networks. Simulation studies demonstrate that TDJGL provides more accurate estimates of gene networks and differential networks than previous competing approaches. We apply TDJGL to the PI3K/AKT/mTOR pathway in ovarian tumors to build differential networks associated with platinum resistance. The hub genes of our inferred differential networks are significantly enriched with known platinum resistance-related genes and include potential platinum resistance-related genes.

Complex biological processes often require the precise regulation and interaction of thousands of genes and their products^1^. For example, in the PI3K/AKT/mTOR pathway, PI3K phosphorylates and activates AKT, and AKT can activate CREB, inhibit p27, localize FOXO in the cytoplasm and activate mTOR^2^. These functional dependence (or regulation) relationships between genes constitute a network, namely gene dependency network, where nodes represent genes and edges represent functional dependence between genes. If we take into account the directionality of edges, gene dependency network is often referred as gene regulatory network^3^. It is well established that cancer progression and drug resistance are induced not only by mutations in genes but also by aberrations in gene networks^4^,^5^,^6^. Therefore, inferring gene networks and exploring how theses networks change across different disease states are of great importance for understanding the biological mechanism behind human cancer and drug resistance^7^,^8^,^9^,^10^,^11^,^12^,^13^,^14^,^15^,^16^,^17^.

The accumulation of gene expression profiles from microarrays paves the way for inferring gene networks using computational methods^9^. Among various network inference algorithms, Gaussian graphical models (GGMs) are popular since the edges identified by them represent conditional dependencies (or direct relationships) between genes^18^,^19^. These models assume that the observed data are generated from a multivariate Gaussian distribution. As a consequence, the conditional dependencies between genes can be determined directly from nonzero elements of the inverse covariance (or precision) matrix^20^, where two genes are conditionally dependent given all other genes if and only if the corresponding element of the precision matrix is nonzero. Thus, the network inference problem can be transformed into a sparse precision matrix estimation problem. Maximum likelihood estimation is a natural way to estimate the precision matrix. However, for gene expression data where the number of genes is often larger than the number of samples, the sample covariance matrix is singular and obtaining an accurate estimate of precision matrix is challenging. In this scenario, the graphical lasso (GL) models^21^,^22^,^23^, which use the prior information that many pairs of genes are conditionally independent, have been proposed and widely used in gene network inference.

Dependencies within gene networks often undergo changes between two groups (e.g. of patients) that represent different stress conditions, tissues, and/or disease states^10^,^24^,^25^,^26^. Differential network analysis has recently emerged as a complement to differential expression analysis to identify altered dependencies between genes across different patient groups^24^,^27^,^28^,^29^. The identification of differential network often consists of two steps: (1) construct weighted group-specific networks using correlation-based methods, where the weights represent the strengths of dependencies; (2) infer differential networks by edge-wise substraction of the strengths of dependencies in the group-specific networks. Here a group-specific network represents the network inferred from a specific group of patients. Although these approaches have successfully addressed some biological problem, they are limited to correlation networks which include both direct and indirect relationships^3^,^30^. In addition, the group-specific networks are estimated separately using observations from each group without considering the fact that there exists some global dependencies that preserve across all groups^29^. As a motivating example, we consider gene networks constructed using gene expression profiles from patients with same type of cancer but different drug responses, such as drug sensitivity and drug resistance. One would expect the two patient group-specific networks to be similar to each other, since both of them are based on the same type of cancer, but also have important differences stemming from the fact that the two groups have different responses to drugs. Estimating the two group-specific networks separately does not exploit the similarity between the true networks, and thus might lead to poor estimates of differential network.

Advances in biotechnology allow biomedical researchers to collect a wide variety of gene expression measurements for the same patients from different platforms^31^. Data repositories such as The Cancer Genome Atlas (TCGA)^32^ have provided gene expression profiles collected from multiple platforms. For instance, TCGA has collected gene expression profiles of patients with ovarian cancer from three platforms (e.g., Agilent 244K Custom Gene Expression G450, Affymetrix HT Human Genome U133 Array Plate Set, and Affymetrix Human Exon 1.0 ST Array). As the multifaceted data are collected for the same patients from distinct but related platforms, they may provide consistent and complement information about the expression level of genes. Therefore, it is of great interest to integrate these data to obtain more accurate and reliable estimations of gene dependency networks and differential networks. Most of previous graphical lasso models consider each platform separately, ignoring the common characteristics shared by different platforms. New statistical models that can borrow strength from different platforms to jointly estimate multiple networks are needed.

In statistics, researchers have proposed several joint graphical lasso (JGL) models to simultaneously estimate multiple related networks using gene expression profiles with observations belongs to distinct groups^25^,^33^,^34^. Compared to graphical lasso^21^,^22^,^23^, the JGL models can improve the accuracy of the resulting networks by considering the common structures preserved across all groups. However, the JGL models assume the group-specific gene expression data are collected from a single platform, which are limited when we have data collected from multiple platforms (Fig. 1(a)). In this setting, we need to model each platform separately if we use the JGL models to jointly infer multiple networks corresponding to different patient groups. This can be suboptimal since the common structures across different patient groups and different platform types cannot be considered simultaneously.

To address the above problems, we propose a two dimensional joint graphical lasso (TDJGL) model to simultaneously infer gene dependency networks corresponding to different patient groups based on gene expression data collected from different platforms (Fig. 1). Our model is an extension of the JGL models to the case where gene expression profiles are characterized in terms of two aspects: patient groups and platform types. It borrows strength across different patient groups and different platform types via a joint penalty function. After obtaining the gene networks, the differential networks between the two patient groups are constructed by calculating the differences of dependencies between two group-specific networks. In simulation studies, TDJGL recovers the true networks and differential networks more accurately than previous competing graphical lasso models. To evaluate the performance of TDJGL on real biological data, we apply it to the challenging problem of identifying differential network associated with platinum response in ovarian cancer. We find the hub genes of the differential networks identified in the PI3K/AKT/mTOR pathway play an important role in cancer drug resistance. The R package of our algorithm is available at https://github.com/Zhangxf-ccnu/TDJGL.

## Methods

### Brief review of Gaussian graphical models and graphical lasso models

Graphical models can encode the conditional dependencies among a set of genes using a graph, where nodes represents genes and edges connect conditionally dependent pairs of genes. A pair of genes are conditionally independent given all the other genes if and only if there is no edge between them^20^. Suppose that we have *n* observations that are independently drawn from a multivariate normal distribution *N*(0, Θ^−1^), where Θ = Σ^−1^ denotes the precision matrix and Σ denotes the covariance matrix. According to the theory of Gaussian graphical models, conditional dependencies among the variables can be directly read from Θ = [*θ*_*ij*_]. In particular, the partial correlation between genes *i* and *j* can be computed as 
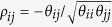
. Therefore, the *i*th and *j*th genes are conditionally independent if and only if *θ*_*ij*_ = 0.

We can estimate Θ via maximum likelihood. However, when the number of genes is larger than the number of observations, this approach fails since the sample covariance matrix is singular. To deal with this problem, graphical lasso, which maximize a penalized log-likelihood, has been proposed^21^,^22^,^23^:


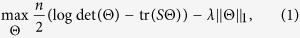


where *S* is the sample covariance matrix, *λ* is a nonnegative tuning parameter, ||Θ||_1_ denotes the sum of the absolute values of the elements of Θ, det(⋅) is the determinant of a matrix and tr(⋅) is the trace of a matrix. The solution to problem (1) serves as a sparse estimate of precision matrix and can be directly used to infer conditional dependencies among genes.

### Problem definition

**Figure 1:**
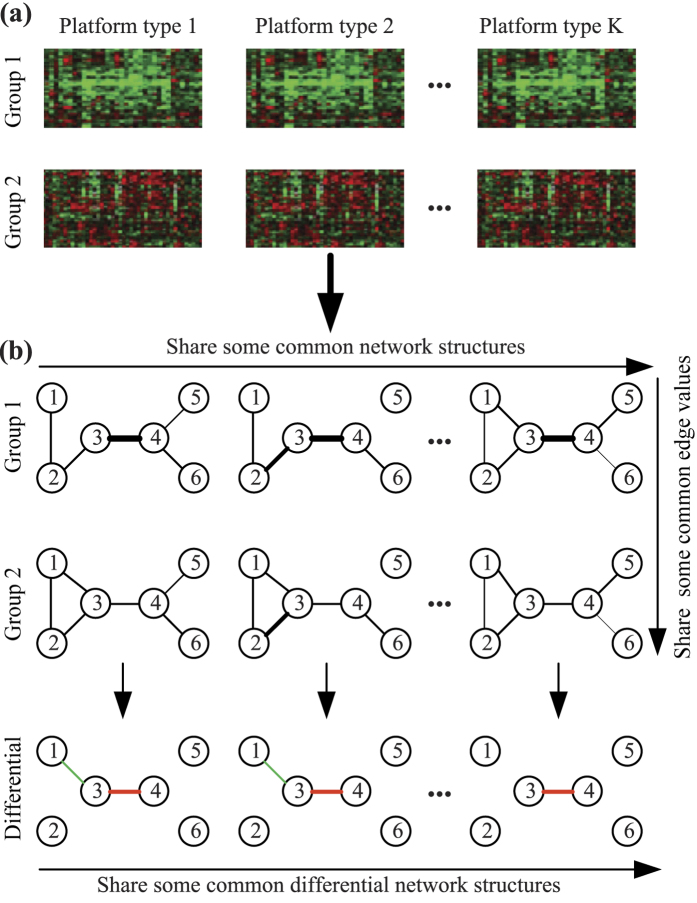
An overview of TDJGL in a toy application to gene network inference and differential network analysis. (**a**) The input data are gene expression profiles for two patient-specific groups collected from *K* platforms. (**b**) TDJGL jointly infers 2*K* conditional dependence networks by borrowing information across the two patient groups and the *K* platform types. Then *K* differential networks are constructed by edge-wise substraction of the dependencies between the group-specific networks. TDJGL encourages the inferred networks to share some common structures. It also encourages identical edge values corresponding to different patient groups for each platform type and same locations of differential edges across the *K* platform types. The red (green) edges indicates positive (negative) differential scores. Edge width is proportional to edge strength.

In this study, we focus on exploring the changes of gene dependency networks between two different patient groups, based on data sets collected from different platforms. Suppose we have collected group-level sample information regarding whether a patient belongs (in general) to group 1 or 2 and gene expression profiles of these samples from multiple microarray platforms (Fig. 1(a)). Our goal is to construct patient group-specific gene networks that present the conditional dependencies among genes for all platforms (Fig. 1(b)). Then, we aim to construct differential networks by identifying conditional dependencies that change under the two patient-specific groups.

### Two dimensional joint graphical lasso model

In this section, we propose a two dimensional joint graphical lasso (TDJGL) model to infer gene networks, which jointly estimates multiple graphical models corresponding to distinct but related platform types and patient groups. We refer to our model as TDJGL since it characterizes the gene expression profiles from two aspects: platform types and patient groups (Fig. 1).

We assume that there are 2*K* data sets 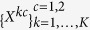 which represent gene expression measurements for 2 patient groups collected from *K* platforms. Here *X*^*kc*^ is a *n*_*c*_ × *p* matrix consisting of measurements for *p* genes, which are common to all 2*K* data sets, from the *k*-th platform on *n*_*c*_ patients in the *c*-th group. Furthermore, we assume that the *n*_1_ + *n*_2_ observations are independent, and that the *n*_*c*_ observations within each data set are from the same Gaussian distribution: 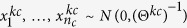, where Θ^*kc*^ is the precision matrix. We seek to estimate the 2*K* precision matrices 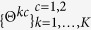 corresponding to the *K* platforms and the 2 patient groups given the 2*K* gene expression data sets. We shall index elements of precision matrix by using *i* = 1, …, *p* and *j* = 1, …, *p*, index platform types by using *k* = 1, …, *K* and index patient groups by using *c* = 1, 2.

Let *S*^*kc*^ = (1/*n*_*c*_)(*X*^*kc*^)^*T*^*X*^*kc*^ be the sample covariance matrix for the *k*-th platform type and the *c*-th patient group. Without loss of generality, here we assume that the observations within each data set are centered. For the sake of convenience, we denotes 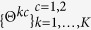 as {Θ}. The negative log-likelihood for the data can be written as^25^,^26^


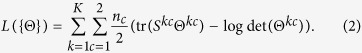


Here we assume that the measurements of the same samples from different platforms are independent for simplicity.

Minimizing Equation (2) with respect to {Θ} yields the maximum likelihood estimates 
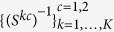
. However, in high dimensional case, the sample covariance matrices are not invertible. Moreover, because the 2*K* data sets correspond to gene expression measurements collected from distinct but related platform types and patient groups, the 2*K* precision matrices may be similar with each other or share some common structures. Therefore, we can combine the 2*K* data sets to estimate the 2*K* precision matrices jointly, rather than estimate them separately.

Following the joint graphical lasso models^25^, instead of estimating precision matrices by minimizing Equation (2), we propose a new penalized log-likelihood based model:


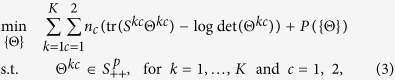


where 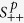 denotes the sets of positive definite matrices of size *p*, and *P*({Θ}) is a penalty function.

Motivated by the property that the number of links in a biological network is far less than that of a full connected network, we require the resulting precision matrices to be sparse. Since the gene expression profiles are collected using similar platforms from related patients, the sparse structure should be preserved across the 2*K* data sets. For each platform, the difference between patient group-specific precision matrices should be sparse. Based on this restriction, we can identify individual edges that are shared or differ across the two patient groups. To incorporate the similarity between different platforms, the sparse structure of differential networks should be preserved across all the *K* platforms. In particular, we develop the following penalty function:


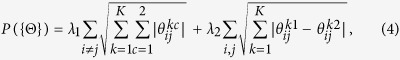


where *λ*_1_ and *λ*_2_ are non-negative tuning parameters. The first term applies a group bridge penalty^35^ to the (*i*, *j*) element across all 2*K* precision matrices where for each pair of genes (*i*, *j*), we treat the 2*K* parameters 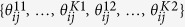 as a group. Here we use the group bridge penalization since it can perform variable selection at both the group and within-group individual variable levels^35^. Therefore, the first term simultaneously encourages a similar pattern of sparsity across all precision matrices and identify both shared edges and data-specific edges across the 2*K* data sets^26^,^36^. The second term applies a group bridge penalty to the (*i*, *j*) element across all the *K* differential networks where for each pair of genes (*i*, *j*), the differences of precision matrices between patient groups across different platforms, 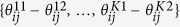, are treated as a group. This bridge group penalty encourages a similar pattern of sparsity across all of the *K* differential networks. Note that here we can also use the group lasso penalty^37^ which has been used in previous studies^25^. We consider the group bridge penalty since it allows the estimated networks to vary across conditions and outperforms the group lasso penalty^26^. The choice of *λ*_1_ and *λ*_2_ controls the sparsity of resulting gene networks and differential networks, which require tuning. We present our parameter selection strategy at the end of this section.

Unlike previously developed joint graphical lasso models^25^,^26^,^34^,^36^ where the data sets are assumed to vary in one dimension, the proposed TDJGL model can borrow strength from two dimensions: platform type and patient group. Since the goal of this study is to identify differential networks between two patient groups, TDJGL encourages identical elements of precision matrices corresponding to the two patient groups, that is, TDJGL penalizes differences between patient groups but not platforms. An alternative to this problem is to penalizes differences between both patient groups and platforms. For our problem, since different data platforms might reflect the dependencies between genes in different scale, it is more reasonable to assign an identical pattern of non-zero elements than to assign identical values across the *K* platforms.

### Algorithm for parameter estimation

We use an iterative approach based on local linear approximation^36^,^38^ to optimize problem (3). Letting 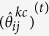 denotes the estimates from the previous iteration *t*, the penalty function (4) can be approximated as


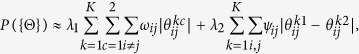


where 
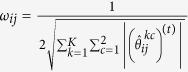
 and 
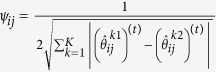
. Thus, at current iteration, problem (3) can be decomposed into *K* individual optimization problems:


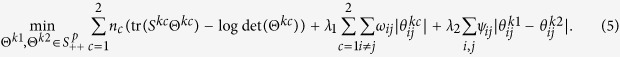


Problem (5) is similar to the fused graphical lasso problem^25^. However, (5) uses a weighted lasso penalty and a weighted fused lasso penalty while the fused graphical lasso model uses a general lasso penalty and a general fused lasso penalty. The weights *ω*_*ij*_ and *ψ*_*ij*_ in (5) are applied to all the *K* platforms, therefore, our model can encourage a shared pattern of network structures across all platforms. Problem (5) can be solved efficiently by using an alternating direction method of multipliers (ADMM)^39^. Due to the lack of space, the details for ADMM algorithm are presented in Supplementary Section S2.2. In summary, the computational algorithm for solving (3) is:

1. Initialize 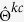 for *k* = 1, …, *K* and *c* = 1, 2.
2. Update 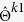 and 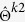 for all *k* = 1, …, *K* by solving problem (5).
3. Repeat Step 2 until convergence is achieved.

Since the penalty function (4) is nonconvex, our algorithm only guarantees to find a local solution. Therefore, the initial value is important to yield an appropriate estimate^26^. When *n*_*c*_ ≥ *p*, we can use (*S*^*kc*^ + *δI*_*p*_)^−1^ as an initial estimate, where *I*_*p*_ is the identity matrix and *δ* is chosen to be a small constant to guarantee *S*^*kc*^ + *δI*_*p*_ is positive definite. Here we set *δ* = 10^−3^. When *n*_*c*_ < *p*, this method does not perform well. In this case, we can use the solution of (5) with *ω*_*ij*_ = 1/2 and *ψ*_*ij*_ = 1/2, because in high dimensional case, a reasonable estimate can be obtained by using a fused graphical lasso model. Our algorithm requires specification of a convergence criterion. Here we declare convergence when


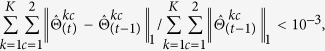


where 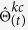 denotes the estimate of Θ^*kc*^ at the *t*th iteration.

### Differential network construction

Through the above algorithm, we obtain the estimates, 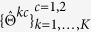, of the 2*K* precision matrices. Conditional dependencies among genes can be directly inferred from the nonzero elements of the estimated precision matrices. That is, genes *i* and *j* are connected in the network for *k*-th platform type and *c*-th patient group if and only if 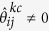. Then, we construct *K* differential networks for different platforms by comparing partial correlations between the two patient groups. For the *k*-th platform type and *c*-th patient group, the partial correlation between genes *i* and *j* can be computed as 
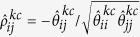
. For the *k*-th platform type, we construct differential score between genes *i* and *j* as 
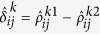
. The absolute value of 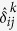 can represent the strength of change, where a larger value indicates a larger change of partial correlation. The sign of 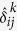 can represent the direction of change, where a positive value represents that the partial correlation is increased in the first patient group compared to the other patient group, while a negative value indicates that the correlation is decreased. The differential scores can be used to construct the differential networks. The presence or absence of edges in the *k*-th differential network is determined by 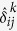: an edge (*i*, *j*) is presented in the *k*-th differential network if and only if 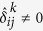. For edges in a differential network, we consider two components: (1) the strength of differential score: 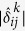, and (2) the sign of differential score: 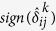. Edges that exist in all the *K* differential networks can be considered as common structures shared by different platforms.

### Model selection

For TDJGL, the tuning parameter *λ*_1_ controls the sparsity of the final gene networks. Larger values of *λ*_1_ tend to yield sparser networks and smaller values of *λ*_1_ yield dense networks. The tuning parameter *λ*_2_ controls the sparsity of the resulting differential networks. When *λ*_2_ is larger, more elements of 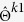 and 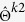 will be identical and the differential networks will be sparser. Therefore, the choice of *λ*_1_ and *λ*_2_ is critical. A number of approaches such as Akaike information criterion, Bayesian information criterion and cross-validation have been used in previous studies. Here we determine the regularization parameters in a data-driven way via stability selection^40^,^41^. Interested reader is referred to Supplementary Section S2.4.

## Results

### Simulation study

In this section, we present the results of simulation experiments that demonstrate the empirical performance of TDJGL.

#### Data generation

In this simulation study, we consider *K* = 3 platform types and 2 patient groups. We generate 6 gene networks (either Erdös-Rényi, scale-free, or community) corresponding to the 3 platform types and the 2 patient groups, each of which contains a common set of *p* genes. For each platform type, we choose *τ* (*τ* = 10%, 20%, 50%) of edges as differential edges between the two patient groups. A larger *τ* represents a larger difference between the two patient groups. The structures of gene networks and differential networks are preserved across the 3 platform types. We generate the Erdös-Rényi, scale-free, and community networks following the settings of Mohan *et al*.^33^ (Supplementary Figures S1). Note that we use a different method to generate differential networks due to different goal. We focus on identifying differential edges, while Mohan *et al*.^33^ pay attention to detecting nodes that drive the differential network.

Data generated for Erdös-Rényi network: We generate the data as follows, for *p* = 100, and *n* ∈ {50, 100, 200}:

1. We generate an Erdös-Rényi network for which each edge is presented with probability 0.02^33^. We then choose (at random) *τ* of edges as differential edges.
2. For *k* = 1, …, *K*, we repeat Steps 3–5 to generate data sets for each platform type.
3. We create a *p* × *p* symmetric matrix *A*^*k*1^ with zeros on elements not corresponding to network edges, and values from Unif([−1, −0.5] ∪ [0.5,1]) on elements corresponding to network edges. We duplicate *A*^*k*1^ into *A*^*k*2^. Then, we set the elements of *A*^*k*2^ corresponding to differential edges to be zeros or change their signs (at random). This results in *τ* of edge values that are different between the two patient groups.
4. We let *d* = min(*λ*_min_(*A*^*k*1^), *λ*_min_(*A*^*k*2^)), where *λ*_min_(⋅) denotes the smallest eigenvalue of the matrix. To ensure positive definiteness, we set Θ^*k*1^ = *A*^*k*1^ + (0.1 + |*d*|)*I*_*p*_ and Θ^*k*2^ = *A*^*k*2^ + (0.1 + |*d*|)*I*_*p*_.
5. We generate *n* independent observations each from a *N*(0, (Θ^*k*1^)^−1^) distribution and a *N*(0, (Θ^*k*2^)^−1^) distribution, and use them as gene expression data sets *X*^*k*1^ and *X*^*k*2^.

Data generated for scale-free network: The data are generated as Erdös-Rényi network, expect that the network generation process in Step 1 is modified: Instead of generating an Erdös-Rényi network, we use the SFNG function in Matlab with parameters *mlinks* = 2 and *seed* = 1 to generate a scale-free network with *p* = 100 genes^33^.

Data generated for community network: We generate data as Erdös-Rényi network, expect for one modification in Step 3: After obtaining *A*^*k*1^ and *A*^*k*2^, the [1:40, 61:100] and [61:100, 1:40] submatrices of *A*^*k*1^ and *A*^*k*2^ are set equal to zero. That is, the non-zero elements of *A*^*k*1^ and *A*^*k*2^ are concentrated in the top and bottom 60 × 60 submatrices^33^. The top and bottom 60 genes correspond to two communities, and genes 40:60 are shared by the two communities.

### Simulation results

We use several metrics to evaluate algorithm performance. We are interested in quantifying (1) recovery of edges, (2) detection of differential edges, and (3) error in estimation of precision matrices. Details are presented in Table 1. We compare the performance of TDJGL to graphical lasso (GL)^22^ and two joint graphical lasso (JGL) models that jointly estimate multiple precision matrices: fused graphical lasso (FGL)^25^ and group graphical lasso (GGL)^25^. FGL is based on the assumption that the difference between precision matrices is sparse, and GGL encourages a similar pattern of sparsity across all of the precision matrices. When applying GL, we fit networks for each platform type and each patient group separately. When applying FGL, networks are fitted for each platform type separately. That is, given a platform type, we fit 2 networks for the two patient groups using FGL. When applying GGL, we fit networks for each patient group separately. For TDJGL, we fit the 6 networks simultaneously. For GGL, we reparameterize the tuning parameters as suggested by Danaher *et al*.^25^, 
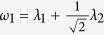
 and 
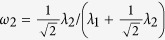
.

**Figure 2:**
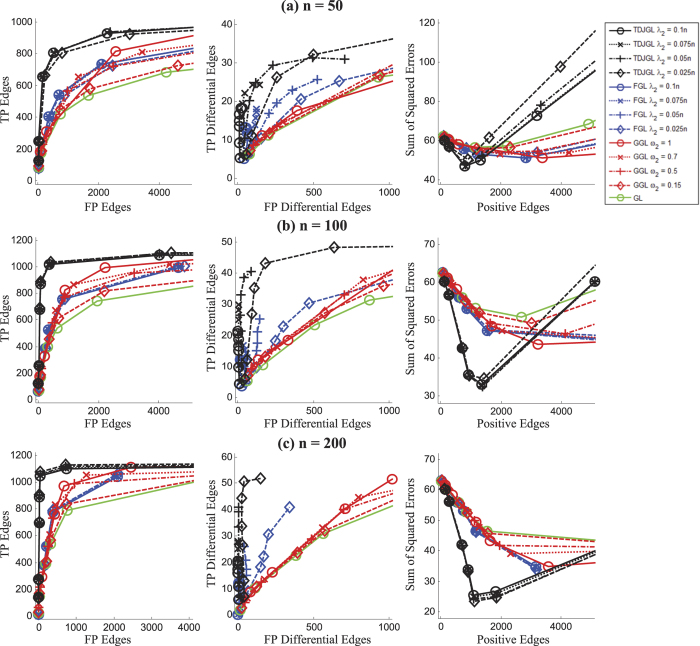
Performance of the compared models on scale-free network with *p* = 100, *K* = 3, *τ* = 10% and (**a**) *n* = 50, (**b**) *n* = 100, (**c**) *n* = 200. Each colored line corresponds to a fixed value of *λ*_2_ (*ω*_2_ for GGL), as *λ*_1_ (*ω*_1_ for GGL) is varied. Variables corresponding to the axes are explained in Table 1. Results are averaged over 100 random generations of the data.

Figure 2 displays the average performance of the compared approaches on scale-free network (with *τ* = 10%) over 100 random generations of the data. Each row corresponds to a sample size and each column corresponds to a performance metric. Within each plot, each colored line corresponds to the results obtained using a fixed value of the tuning parameter *λ*_2_ (for TDJGL and FGL) or *ω*_2_ (for GGL), as the tuning parameter *λ*_1_ (for TDJGL and FGL) or *ω*_1_ (for GGL) is varied. Note that GL corresponds to FGL with *λ*_2_ = 0 or GGL with *ω*_2_ = 0. We observe that TDJGL outperforms the three compared methods for a suitable range of the parameters *λ*_2_ and *ω*_2_. For a fixed number of false positive edges, TDJGL identifies more true positive edges; for a fixed number of false positive differential edges, TDJGL identifies a greater number of true positive differential edges; and for a fixed number of edges estimated, TDJGL has a lower squared error. Unlike FGL which only exploits similarity between the two patient groups and GGL which only borrows strength across different platform types, TDJGL is capable of making full use of the characteristics shared by different platform types and different patient groups. FGL and GGL have similar performance when we focus on identifying edges and estimating precision matrices. However, FGL dominates GGL when it is used to identify differential edges, since it shrinks the difference between edge values corresponding to two different patient groups. GL perform worst among the four methods, since it estimates each network separately. The simulation results for the Erdos-Renyi and community networks (with *τ* = 10%) are displayed in Supplementary Figures S2 and S3, respectively. We also present the results for scale-free network (with *τ* = 20%, 50%) in Supplementary Figures S4 and S5. These results also show that TDJGL substantially outperforms the state-of-the-art methods.

## TCGA ovarian cancer application

In this section, we apply TDJGL to analyze gene expression data of ovarian cancer and present the corresponding results.

### Data sets

Ovarian cancer is the most common cause of death from gynaecological cancers, and overall survival has not improved significantly for several decades^42^. One factor that accounts for treatment failure and high mortality associated with ovarian cancer is treatment resistance^42^,^43^. To successfully treat ovarian cancer and improve overall survival, we need to overcome the development of resistance to platinum chemotherapy. In order to obtain a better understanding of the underlying mechanism of platinum resistance, we are interested in determining how the gene dependency networks are changed between ovarian tumors with different treatment responses (platinum-sensitive and platinum-resistant). We apply TDJGL to gene expression data from TCGA, which are collected from three platforms: Agilent 244K Custom Gene Expression G450, Affymetrix HT Human Genome U133 Array Plate Set, and Affymetrix Human Exon 1.0 ST Array^32^. For the sake of convenience, we refer to them as G450, U133 and HuEx, respectively. We download these gene expression profiles (level 3) from the TCGA website. As of February 2016, gene expression levels of 11,750 genes for 514 patients across all the three platforms are available. We then take a logarithmic transformation to make the data more normally distributed.

We use a criterion that is used in refs 32 and 44 to define platinum-based chemotherapy response groups: platinum-sensitive and platinum-resistant. Tumors are defined as platinum-sensitive if there is no evidence of disease progression within 6 months of the end of the last primary treatment, and the follow-up interval is at least 6 months from the date of last primary treatment. Tumors with evidence of disease progression within 6 months of the end of primary treatment are defined as platinum-resistant (For detail, refer to Supplementary Section S2.5). Among the 514 tumors, 340 tumors are identified with explicit cis-platinum status, with 242 platinum-sensitive tumors and 98 platinum-resistant tumors. The sensitive and resistant information for each sample is presented in Supplementary information 2. For each platform, the gene expression data sets are standardized to have mean 0 and standard deviation 1 within each patient group. The gene expression data for the 340 tumors which have cis-platinum status are provided at https://github.com/Zhangxf-ccnu/TDJGL.

To make the computation less intensive, we take a pathway-based analysis. We present our analysis of genes that overlap with the PI3K/AKT/mTOR pathway. The PI3K/AKT/mTOR pathway is frequently mutated or altered in ovarian cancer^32^, and is often implicated in resistance to anticancer therapies^45^. We download the PI3K/AKT signaling pathway and the mTOR signaling pathway from the Kyoto Encyclopedia of Genes and Genomes database^46^. Among the 362 genes in the PI3K/AKT/mTOR pathway, there are 301 genes in our considered gene expression data sets. We hypothesize that the identification of the differential network within the PI3K/AKT/mTOR pathway between platinum-sensitive tumors and platinum-resistant tumors will provide a new understanding of mechanism of drug response.

### Differential networks analysis

We apply TDJGL to gene expression data from the three platforms with respect to platinum-sensitive tumors and platinum-resistant tumors. To avoid disparate level of sparsity between the two patient groups, we weight each patient group equally instead of by sample size in Equation (3) 25. We select parameters *λ*_1_ and *λ*_2_ from a total of 20 possible values equally spaced in log scale between 0.25 and 0.025. According to the StARS model selection approach (Supplementary Section S2.4), we set *λ*_1_ = 0.154 and *λ*_2_ = 0.0406 to yield sparse and stable networks. After obtaining the 6 precision matrices by solving TDJGL, we infer group-specific gene networks and differential networks based on the estimated precision matrices (See the Differential network construction section). The estimated group-specific networks and differential networks are provided in Supplementary information 3.

We observe that most of edges identified by TDJGL are common to both patient groups and there are only a few differential edges for all the three platforms (Supplementary Figure S6). This might owe to the fact TDJGL can borrow information aggressively between the two patient groups to encourage not only similar network structures but also similar edge values. In addition, the overlaps between the edges (and differential edges) detected by TDJGL from the three platforms are substantially large (Supplementary Figure S6), which indicates that our model can encourage a shared pattern of network structures (and differential network structures) across different platforms.

A hub gene within a network is important for the control of the underlying network^47^. Therefore, we are interested in the biological significance of hub genes in the estimated differential networks. Table 2 presents the 18 hub genes that have degrees greater than 2 in all the three differential networks constructed from different platforms. Assuming that hub genes may contribute to cancer drug resistance, we expect that genes associated with drug resistance and genes causally implicated in cancer may significantly appear in the set of hub genes. We collect 161 cisplatin resistance-related genes and 758 drug resistance-related genes from the database of Genomic Elements Associated with drug Resistance (GEAR). Among the 301 genes in the PI3K/AKT/mTOR pathway, there are 26 genes and 74 genes associated with cisplatin resistance and drug resistance, respectively. We also obtain 572 genes for which mutations have been causally implicated in cancer from the Cancer Gene Census (CGC) database^48^, and there are 60 cancer-related genes in the PI3K/AKT/mTOR pathway. We observe that out of the 18 hub genes, 5 of them are cisplatin resistance-related genes, 10 of them are drug resistance-related genes and 8 of them are cancer-related genes (Table 2). According to the Fishers exact test, the set of hub genes is significantly enriched with the three types of biologically important genes (The p-values are 0.0128, 0.0036 and 0.0132, respectively).

**Figure 3:**
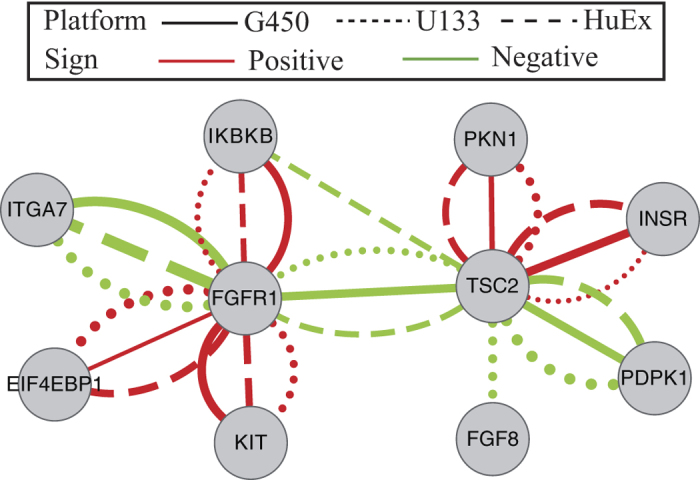
Two hub genes (FGFR1 and TSC2) and their neighbors of differential networks between platinum-sensitive tumors and platinum-resistant tumors inferred by TDJGL in the PI3K/AKT/mTOR pathway. The solid, dot, and long dash lines represents differential edges identified from the G450, U133 and HuEx platforms, respectively. The red (green) edges indicates positive (negative) differential scores. The thickness of the edges correspond to the strengths of dependencies, with strong scores having greater thickness.

Besides well-known genes (e.g., CCNE2, AKT1 and MYC) associated with platinum (or drug) resistance, the other hub genes (e.g., FGFR1 and TSC2) may be potential platinum resistance-related genes. FGFR1 is receptor tyrosine kinase which plays an essential role in the regulation of embryonic development, cell proliferation, differentiation and migration. Amplification of FGFR1 has been reported frequently in ovarian cancer, and is associated with poor survival^49^,^50^. We observe that the dependencies between FGFR1 and other five genes undergo change between the two patient groups (Fig. 3). Among the five neighbors of FGFR1 in the differential networks, two of them (KIT and EIF4EBP1) have been reported to be associated with drug resistance^51^,^52^. In a recent study, Formisano *et al*.^53^ have found that FGFR1 is associated with resistance to endocrine therapy in ER+/FGFR1-amplified breast cancer. TSC2, which connects with FGFR1 in all the three differential networks, is other hub gene (Fig. 3). TSC2 is a tumor suppressor that interacts with TSC1 to control mTOR signaling by regulating mTORC1 activity. Copy number loss and lower expression level of TSC2 have been observed in primary ovarian serous tumors^54^. One of its neighbor in the differential networks, PDK1, is a critical oncogene in ovarian serous carcinoma^55^ and is associated with chemoresistance^56^. In particular, Wagle *et al*.^57^ have recently revealed that mutation in TSC2 is associated with sensitivity to everolimus in anaplastic thyroid cancer. Therefore, it is our hypothesis that FGFR1 and TSC2 might be associated with platinum resistance in ovarian cancer. None of them are identified as genes associated platinum resistance in previous differential gene analysis^44^. Thus, it is of interest to study how the dependencies between the two hub genes and their neighbors correlate with platinum response in ovarian cancer.

We present the additional comparison of TDJGL with other graphical models (GL^22^, FGL^25^ and GGL^25^) with application to ovarian cancer gene expression data in Supplementary Section S2.6. Experiment results indicate that TDJGL outperforms the competing models in terms of the overlap between edges (and differential edges) identified from different platforms and the functional significance of hub nodes in the inferred differential networks.

## Discussion

We have proposed TDJGL, a method for inferring patient group-specific gene networks and identifying differential networks between two patient-specific groups from gene expression data collected from different platforms. TDJGL jointly estimates multiple conditional dependence networks corresponding to different but related patient groups and platform types. It borrows strength across different data sets through a joint sparsity penalty function. TDJGL outperforms several competing algorithms over a range of simulated data sets. We apply TDJGL to TCGA ovarian cancer gene expression data from three platforms to identify differential networks associated with platinum resistance. In the PI3K/AKT/mTOR pathway, the set of hub genes in the estimated differential networks is significantly enriched with drug resistance-related genes and cancer-related genes. The hub genes (e.g., FGFR1 and TSC2) which have not been reported in previous literature might be potential platinum resistance-related genes in ovarian cancer.

In previous studies, joint graphical lasso models have been proposed to estimate multiple gene networks from observations belonging to different patient-specific groups. However, these studies only focus on gene expression data from single platform. Advances in high-throughput technologies allow us to collect gene expression measurements on a common set of samples from multiple platforms. TDJGL infer gene networks for different patient groups by integrating gene expression profiles collected from different platforms. Unlike previous joint graphical lasso models which can only borrow strength from one aspect (e.g., patient groups), TDJGL is a new extension to borrow information from two aspects (e.g., patient groups and platform types).

In general, it is time-consuming and difficult for graphical lasso-based models to scale up^58^. This is because most of learning algorithms need to compute the eigendecomposition of a *p* × *p* matrix in the ADMM iteration, where *p* is the number of genes (Supplementary Section S2.2). Thus, we take a pathway-based analysis in this study. In particular, we pay our attention to the PI3K/AKT/mTOR pathway since it plays an important role in cancer drug resistance. The goal of this paper is to propose a new statistical model to estimate differential networks from gene expression data collected from multiple platforms. Therefore, we do not analyze other pathways. Interested reader can use our R package to analyze other pathways. In order to fit genome-wide data, we will extend TDJGL to consider the pathway-based constraints, following the method of pathway graphical lasso^58^. In addition, we will consider speed-ups of our local linear approximation and ADMM algorithms as well as the usage of other fast algorithms such as the accelerated proximal gradient method or second-order methods in future work.

Our study may be extended in the following aspects. In this study, TDJGL is applied to the microarray gene expression data measured on multiple platforms and two patient groups. However, our model can be equally applicable to repeated measures using the same platform on two patients groups. TDJGL assumes the data is generated from a Gaussian distribution. This assumption only holds for microarray-based gene expression data. As RNA-seq quantification is based on read counts, the Gaussian distribution assumption is unsuitable for data from RNA-seq experiments, which are often modeled as negative binomial or Poisson distributed^59^,^60^. Therefore, our model is limited to microarray data and is not optimal for RNA-seq data. It is of interest to extend our method to fit RNA-seq data following the method of Poisson graphical models^61^,^62^. In this study, we infer gene networks using gene expression data collected from different platforms. Besides gene expression data, TCGA also provides gene-level activity measurements generated by other omics technologies (e.g., methylation and copy number). Different omics data include both homogeneous and heterogeneous information. We will consider how to extend our model to integrate multi-omics data to infer gene networks and identify differential networks between different patient-specific groups. TDJGL has potential applications beyond those discussed in this study. For instance, it can be used in Gaussian model-based clustering to reduce the variance, and further used to reveal cancer subtypes^63^.

## Additional Information

**How to cite this article**: Zhang, X.-F. *et al*. Differential network analysis from cross-platform gene expression data. *Sci. Rep.* **6**, 34112; doi: 10.1038/srep34112 (2016).

## Supplementary Material

Supplementary Information 2

Supplementary Information 3

Supplementary Information 1

## Figures and Tables

**Table 1:** Metrics used to quantify algorithm performance.

(1)	Positive edges: 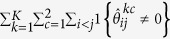
	True positive (TP) edges: 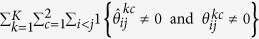
	False positive (FP) edges: 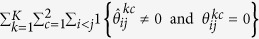
(2)	True positive differential edges: 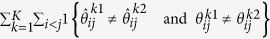
	False positive differential edges: 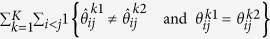
(3)	Error: 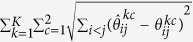

Here 1{*A*} is an indicator variable that equals to one if the event *A* holds and equals zero otherwise.

**Table 2:** List of hub genes of differential networks detected by TDJGL from the PI3K/AKT/mTOR pathway.

Genes	Degree	GEAR_cisplatin_	GEAR_drug_	CGC
CCNE2	7|6|7		×	
AKT1	5|5|5	×	×	×
FGFR1	5|5|5			×
MYC	5|4|5	×	×	×
TSC2	4|5|5			×
BCL2L1	5|3|5	×	×	
INSR	4|4|4			
KIT	4|4|4		×	×
PPP2R2B	4|4|4		×	
CCND2	4|4|3		×	×
LAMB3	3|4|4			
CDKN1A	4|3|3	×	×	
GNG12	3|3|4			
HGF	3|4|3		×	
CASP9	3|3|3	×	×	
CCNE1	3|3|3			×
EIF4B	3|3|3			
MTCP1	3|3|3			×

If a gene is associated with resistance to cisplatin (GEAR_cisplatin_) and resistance to drug (GEAR_drug_) according to the database of Genomic Elements Associated with drug Resistance, and causally implicated in cancer (CGC) according to the Cancer Gene Census database, there is an × in the corresponding entry. *a*|*b*|*c*^§^ denotes the degree of genes in differential networks constructed from the G450, U133 and HuEx platforms, respectively.
