# Supplementary material for: Differential network analysis from cross-platform gene expression data
Source: Sci Rep. 2016 Sep 28;6:34112. doi: 10.1038/srep34112 (PMC5039701; doi:10.1038/srep34112)
Supplement: Supplementary Information 1 [file srep34112-s3.pdf]

Differential network analysis from cross-platform gene expression data: Supplementary Information

Xiao-Fei Zhang, Le Ou-Yang, Xing-Ming Zhao, and Hong Yan

Contents

1 Supplementary Figures **2**

2 Supplementary Text **7**

2.1 Brief review of ADMM algorithms 7

2.2 ADMM algorithm for fused graphical lasso with weighted ℓ_1 penalty 7

2.2.1 $\mathbf{\Theta}^{k1}$ and $\mathbf{\Theta}^{k2}$ update 8

2.2.2 $\mathbf{Z}^{k1}$ and $\mathbf{Z}^{k2}$ update 8

2.2.3 Complete ADMM algorithm 8

2.2.4 Stop criteria 8

2.2.5 Varying penalty parameter 9

2.3 Complete algorithm of the TDJGL model 9

2.4 Model selection 10

2.5 Criteria for platinum response groups 11

2.6 Comparison with other graphical lasso models on the ovarian cancer data 11

1 Supplementary Figures

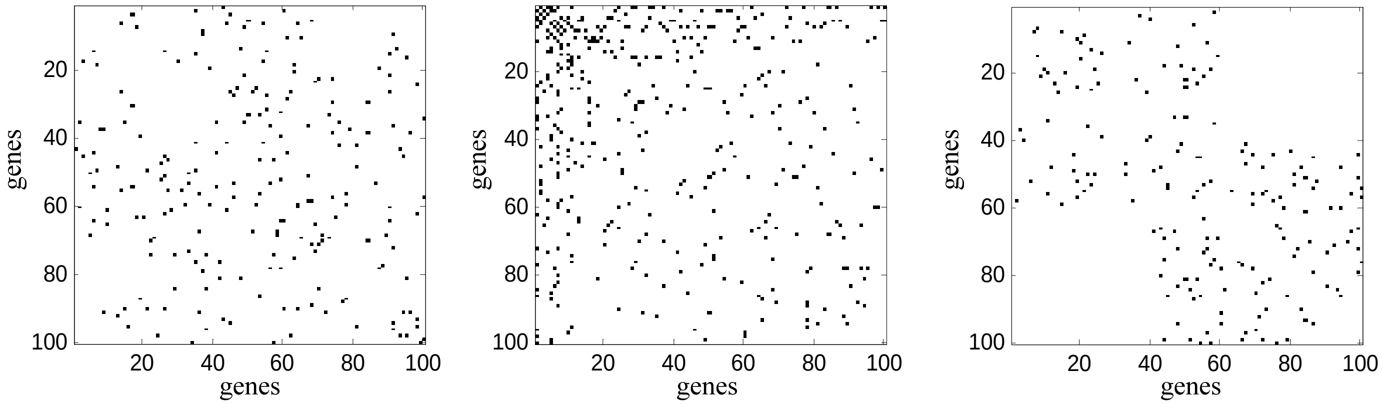

(a) Erdös-Rényi network (b) scale free network (c) community network

Figure S1: Examples for three types of networks used to generate simulated data sets. (a) Erdös-Rényi network, (b) scale-free network, and (c) community network.

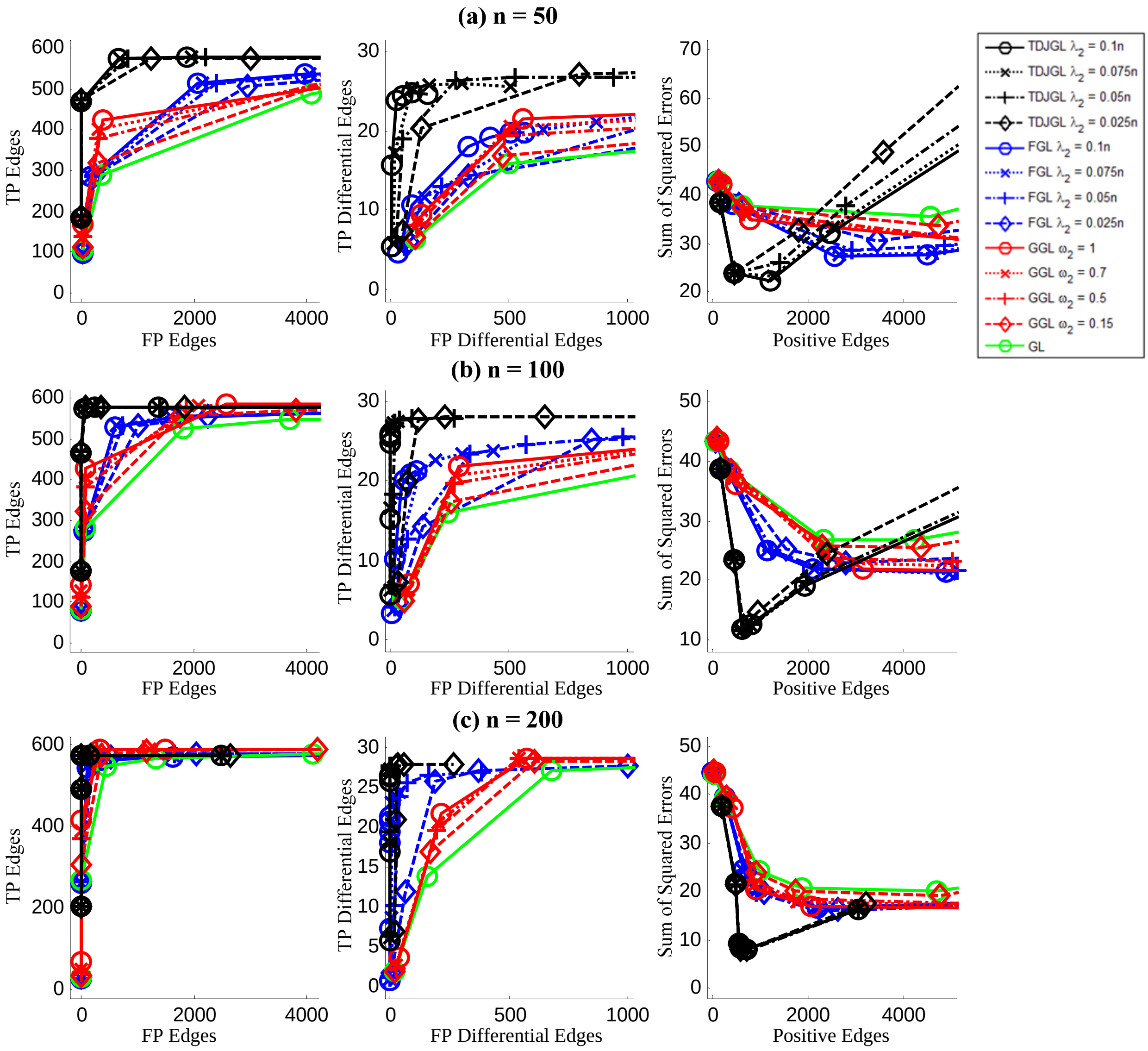

Figure S2: Performance of the compared methods on Erdos-Renyi network with $p = 100$, $K = 3$, $\tau = 10\%$ and (a) $n = 50$, (b) $n = 100$, (c) $n = 200$. Each colored line corresponds to a fixed value of λ_2 (ω_2 for GGL), as λ_1 (ω_1 for GGL) is varied. Variables corresponding to the axes are explained in Table 1 in the main text. Results are averaged over 100 random generations of the data.

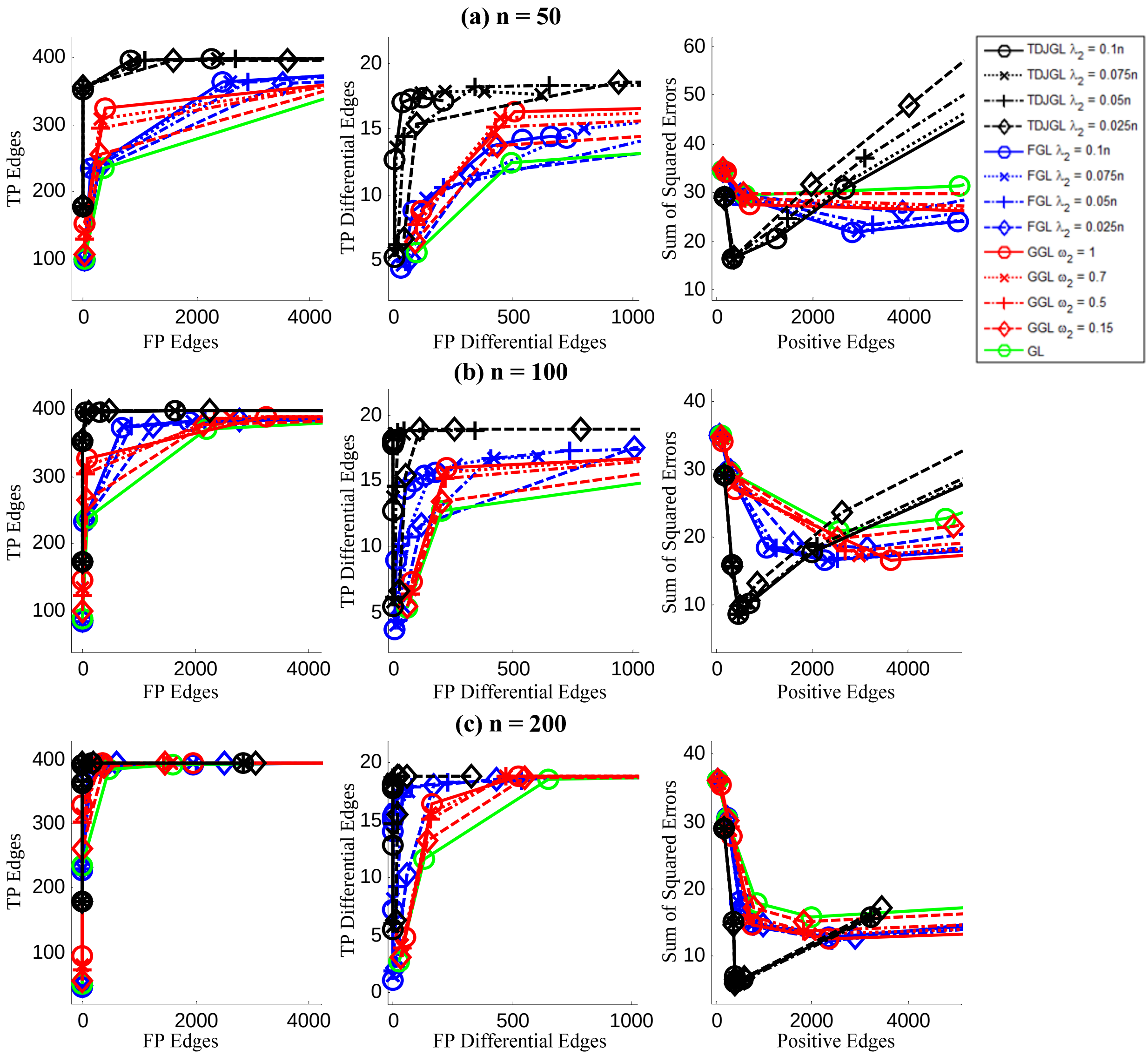

Figure S3: Performance of the compared methods on community network with $p = 100$, $K = 3$, $\tau = 10\%$ and (a) $n = 50$, (b) $n = 100$, (c) $n = 200$. Each colored line corresponds to a fixed value of λ_2 (ω_2 for GGL), as λ_1 (ω_1 for GGL) is varied. Variables corresponding to the axes are explained in Table 1 in the main text. Results are averaged over 100 random generations of the data.

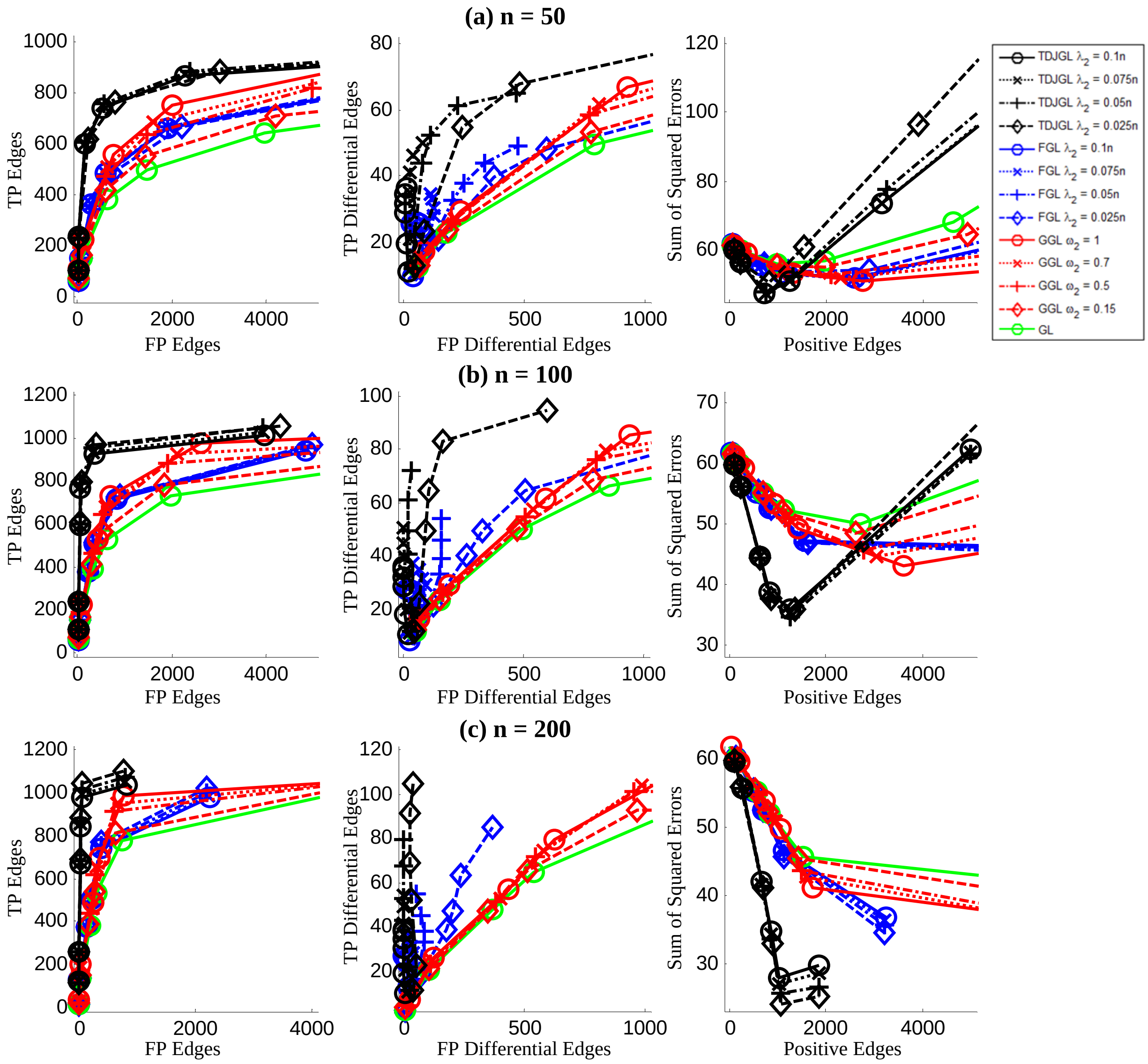

Figure S4: Performance of the compared models on scale-free network with $p = 100$, $K = 3$, $\tau = 20\%$ and (a) $n = 50$, (b) $n = 100$, (c) $n = 200$. Each colored line corresponds to a fixed value of λ_2 (ω_2 for GGL), as λ_1 (ω_1 for GGL) is varied. Variables corresponding to the axes are explained in Table 1 in the main text. Results are averaged over 100 random generations of the data.

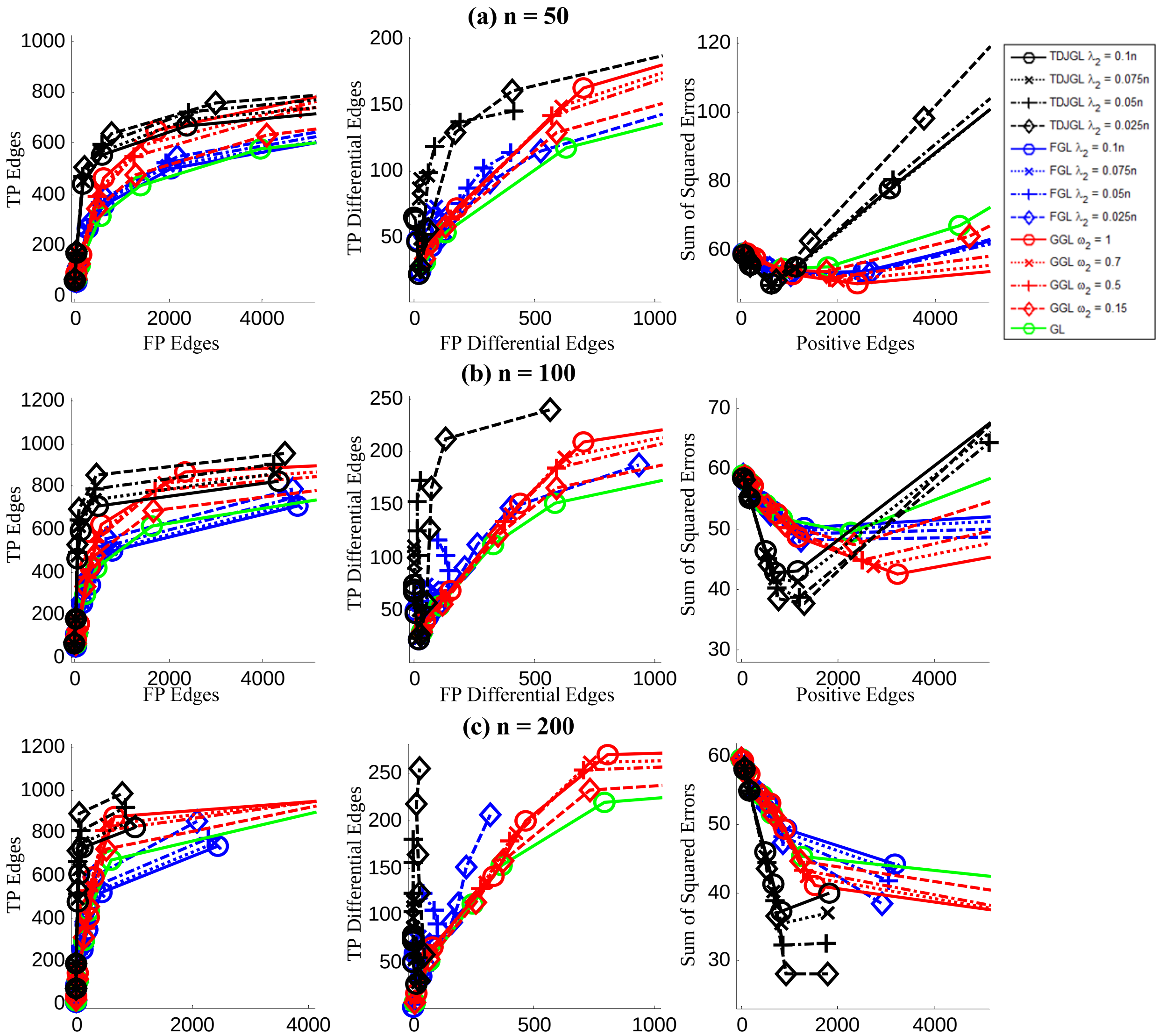

Figure S5: Performance of the compared models on scale-free network with $p = 100$, $K = 3$, $\tau = 50\%$ and (a) $n = 50$, (b) $n = 100$, (c) $n = 200$. Each colored line corresponds to a fixed value of λ_2 (ω_2 for GGL), as λ_1 (ω_1 for GGL) is varied. Variables corresponding to the axes are explained in Table 1 in the main text. Results are averaged over 100 random generations of the data.

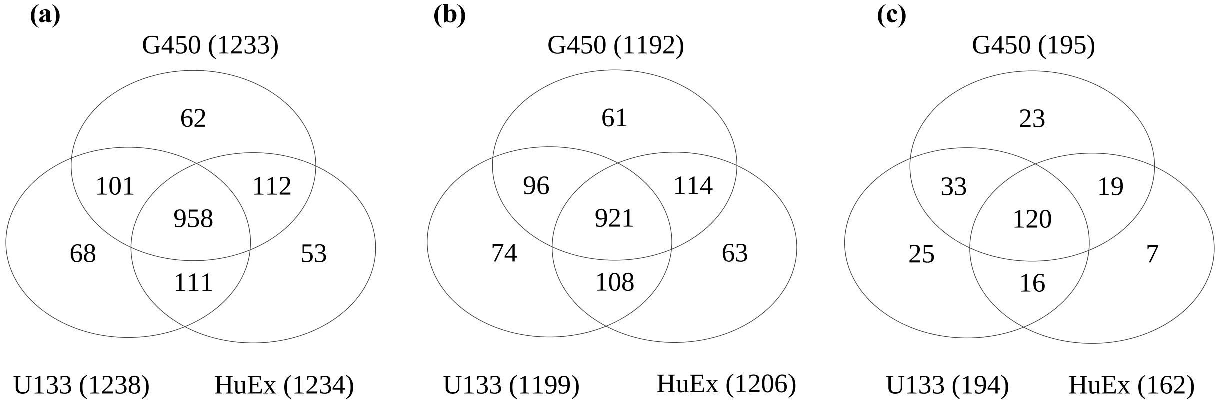

Figure S6: Overlaps between the edges (and differential edges) detected by TDJGL from the three platforms for (a) platinum-resistant tumors, (b) platinum-sensitive tumors and (c) differential networks.

2 Supplementary Text

2.1 Brief review of ADMM algorithms

In this section, we briefly review the standard alternating direction method of multipliers (ADMM) algorithms [1]. ADMM is a technique for solving optimization problem in the following form [2]:

$$\begin{aligned} \min_{\mathbf{X}} \quad & f(\mathbf{X}) + g(\mathbf{X}) \\ \text{subject to} \quad & \mathbf{X} \in \mathcal{X}. \end{aligned} \tag{1}$$

ADMM is attractive when the proximal operator of $f(\mathbf{X}) + g(\mathbf{X})$ cannot be easily obtained, but the proximal operator of $f(\mathbf{X})$ and the proximal operator of $g(\mathbf{X})$ can be easily computed. The approach consists of the following three steps [2]:

1. Rewrite the problem (1) as

$$\begin{aligned} \min_{\mathbf{X},\mathbf{Z}} \quad & f(\mathbf{X}) + g(\mathbf{Z}) \\ \text{subject to} \quad & \mathbf{X} \in \mathcal{X}, \mathbf{X} = \mathbf{Z}, \end{aligned} \tag{2}$$

   where functions f and g are decoupled by introducing a new optimization variable, $\mathbf{Z}$.

2. Form the augmented Lagrangian

$$L_\rho(\mathbf{X}, \mathbf{Z}, \mathbf{\Lambda}) = f(\mathbf{X}) + g(\mathbf{Z}) + \langle \mathbf{\Lambda}, \mathbf{X} - \mathbf{Z} \rangle + \frac{\rho}{2} \|\mathbf{X} - \mathbf{Z}\|_F^2, \tag{3}$$

   where $\mathbf{\Lambda}$ is the Lagrange multiplier and $\rho > 0$ is a penalty parameter.

3. Iterate the following two steps until convergence

   (a) Update each primal variable in turn by minimizing the augmented Lagrangian (3) with respect to that variable, while holding all other variables. The update in the tth iteration are as follows:

$$\mathbf{X}^{t+1} \leftarrow \arg\min_{\mathbf{X} \in \mathcal{X}} L_\rho\left(\mathbf{X}, \mathbf{Z}^t, \mathbf{\Lambda}^t\right),$$

$$\mathbf{Z}^{t+1} \leftarrow \arg\min_{Z} L_\rho\left(\mathbf{X}^t, \mathbf{Z}, \mathbf{\Lambda}^t\right).$$

   (b) Update the dual variable using a dual-ascent update:

$$\mathbf{\Lambda}^{t+1} \leftarrow \mathbf{\Lambda}^t + \rho\left(\mathbf{X}^{t+1} - \mathbf{Z}^{t+1}\right).$$

2.2 ADMM algorithm for fused graphical lasso with weighted ℓ_1 penalty

Following in the method of [3], we use the ADMM algorithm to solve the fused graphical lasso with weighted ℓ_1 penalty (problem (5) in the main text)

$$\min_{\mathbf{\Theta}^{k1},\, \mathbf{\Theta}^{k2} \in S_{++}^p} \sum_{c=1}^{2} n_c \left(\operatorname{tr}\left(\mathbf{S}^{kc}\mathbf{\Theta}^{kc}\right) - \log\det\left(\mathbf{\Theta}^{kc}\right)\right) + \lambda_1 \sum_{c=1}^{2} \sum_{i \neq j} \omega_{ij} \left|\theta_{ij}^{kc}\right| + \lambda_2 \sum_{i,j} \psi_{ij} \left|\theta_{ij}^{k1} - \theta_{ij}^{k2}\right|. \tag{4}$$

To solve the problem (4), we reformulate it by introducing new variables $\mathbf{Z}^{k1}$ and $\mathbf{Z}^{k2}$, so as to decouple some of the terms in the objective function that are difficult to optimize jointly:

$$\begin{aligned} \min_{\mathbf{\Theta}^{k1},\, \mathbf{\Theta}^{k2} \in S_{++}^p,\, \mathbf{Z}^{k1},\, \mathbf{Z}^{k2}} \quad & \sum_{c=1}^{2} n_c \left(\operatorname{tr}\left(\mathbf{S}^{kc}\mathbf{\Theta}^{kc}\right) - \log\det\left(\mathbf{\Theta}^{kc}\right)\right) + \lambda_1 \sum_{c=1}^{2} \sum_{i \neq j} \omega_{ij} \left|z_{ij}^{kc}\right| + \lambda_2 \sum_{i,j} \psi_{ij} \left|z_{ij}^{k1} - z_{ij}^{k2}\right| \\ \text{subject to} \quad & \mathbf{\Theta}^{k1} = \mathbf{Z}^{k1},\ \mathbf{\Theta}^{k2} = \mathbf{Z}^{k2}. \end{aligned} \tag{5}$$

The augmented Lagrangian to (5) is given by

$$\begin{aligned} L_\rho\left(\mathbf{\Theta}^{k1},\, \mathbf{\Theta}^{k2},\, \mathbf{Z}^{k1},\, \mathbf{Z}^{k2},\, \mathbf{U}^{k1},\, \mathbf{U}^{k2}\right) = & \sum_{c=1}^{2} n_c \left(\operatorname{tr}\left(\mathbf{S}^{kc}\mathbf{\Theta}^{kc}\right) - \log\det\left(\mathbf{\Theta}^{kc}\right)\right) + \lambda_1 \sum_{c=1}^{2} \sum_{i \neq j} \omega_{ij} \left|z_{ij}^{kc}\right| \\ & + \lambda_2 \sum_{i,j} \psi_{ij} \left|z_{ij}^{k1} - z_{ij}^{k2}\right| + \left\langle \mathbf{U}^{k1}, \mathbf{\Theta}^{k1} - \mathbf{Z}^{k1} \right\rangle + \left\langle \mathbf{U}^{k2}, \mathbf{\Theta}^{k2} - \mathbf{Z}^{k2} \right\rangle \\ & + \frac{\rho}{2} \left\|\mathbf{\Theta}^{k1} - \mathbf{Z}^{k1}\right\|_F^2 + \frac{\rho}{2} \left\|\mathbf{\Theta}^{k2} - \mathbf{Z}^{k2}\right\|_F^2, \end{aligned} \tag{6}$$

where $\mathbf{U}^{k1}$ and $\mathbf{U}^{k2}$ are dual variables and ρ severs as the penalty parameter.

The ADMM algorithm updates each primal variable while holding the other variables fixed and then updates the dual variables using a dual-ascent update rule. We now derive the update rules for the variables.

2.2.1 Θ^{k1} and Θ^{k2} update

Before introducing the update rules for Θ^{k1} and Θ^{k2}, we first define the Expand operator:

$$\mathrm{Expand}\left(\mathbf{A}, \rho, n\right) = \arg\min_{\Theta \in S_{++}^{p}} -n \log\det\left(\Theta\right) + \frac{\rho}{2}\left\|\Theta - \mathbf{A}\right\|^2 = \frac{1}{2}\mathbf{U}\left(D + \sqrt{D^2 + \frac{4n}{\rho}\mathbf{I}}\right)\mathbf{U}^{\mathbf{T}}, \tag{7}$$

where $\mathbf{UDU^T}$ is the eigenvalue decomposition of a symmetric matrix $\mathbf{A}$. The Expand operator has been used to solve the graphical lasso problem in previous studies [2, 4, 3].

Note that

$$\begin{aligned}\Theta^{k1} &= \arg\min_{\Theta^{k1} \in S_{++}^{p}} L_\rho\left(\Theta^{k1},\ \Theta^{k2},\ \mathbf{Z}^{k1},\ \mathbf{Z}^{k2},\ \mathbf{U}^{k1},\ \mathbf{U}^{k2}\right) \\ &= \arg\min_{\Theta^{k1} \in S_{++}^{p}} -n_1 \log\det\left(\Theta^{k1}\right) + \frac{\rho}{2}\left\|\Theta^{k1} - \left(\mathbf{Z}^{k1} - \frac{1}{\rho}\left(n_1\mathbf{S}^{k1} + \mathbf{U}^{k1}\right)\right)\right\|_F^2 \end{aligned} \tag{8}$$

Now it follows from the definition of the Expand operator that

$$\Theta^{k1} \leftarrow \mathrm{Expand}\left(\mathbf{Z}^{k1} - \frac{1}{\rho}\left(n_1\mathbf{S}^{k1} + \mathbf{U}^{k1}\right), \rho,\ n_1\right). \tag{9}$$

The update for Θ^{k2} can be derived in a similar way:

$$\Theta^{k2} \leftarrow \mathrm{Expand}\left(\mathbf{Z}^{k2} - \frac{1}{\rho}\left(n_2\mathbf{S}^{k2} + \mathbf{U}^{k2}\right), \rho,\ n_2\right). \tag{10}$$

2.2.2 $\mathbf{Z}^{k1}$ and $\mathbf{Z}^{k2}$ update

Minimizing augmented Lagrangian (6) with respect to $\mathbf{Z}^{k1}$ and $\mathbf{Z}^{k2}$ can be written as follows:

$$\begin{aligned}\{\mathbf{Z}^{k1}, \mathbf{Z}^{k2}\} &= \arg\min_{\{\mathbf{Z}^{k1},\ \mathbf{Z}^{k2}\}} L_\rho\left(\Theta^{k1},\ \Theta^{k2},\ \mathbf{Z}^{k1},\ \mathbf{Z}^{k2},\ \mathbf{U}^{k1},\ \mathbf{U}^{k2}\right) \\ &= \arg\min_{\{\mathbf{Z}^{k1},\ \mathbf{Z}^{k2}\}} \frac{\rho}{2}\sum_{c=1}^{2}\left\|\mathbf{Z}^{kc} - \left(\Theta^{kc} + \frac{\mathbf{U}^{kc}}{\rho}\right)\right\|_F^2 + \lambda_1\sum_{c=1}^{2}\sum_{i\neq j}\omega_{ij}\left|z_{ij}^{kc}\right| + \lambda_2\sum_{i,j}\psi_{ij}\left|z_{ij}^{k1} - z_{ij}^{k2}\right| \end{aligned} \tag{11}$$

Now (11) is completely separable with respect to each pair of matrix elements (i, j). That is, we can solve for each (i, j):

$$\{z_{ij}^{k1}, z_{ij}^{k2}\} = \arg\min_{\{z_{ij}^{k1}, z_{ij}^{k2}\}} \frac{\rho}{2}\sum_{c=1}^{2}\left(z_{ij}^{kc} - a_{ij}^{kc}\right)^2 + \lambda_1\sum_{c=1}^{2}\omega_{ij}\left|z_{ij}^{kc}\right| + \lambda_2\psi_{ij}\left|z_{ij}^{k1} - z_{ij}^{k2}\right|, \tag{12}$$

where $a_{ij}^{kc} = z_{ij}^{kc} + \frac{u_{ij}^{kc}}{\rho}$. This is a special case of the fused lasso signal approximator [3, 5] and it has a very simple closed form solution. When $\lambda_1 = 0$, the solution to (12) takes the form

$$\left(z_{ij}^{k1}, z_{ij}^{k2}\right) = \begin{cases} \left(a_{ij}^{k1} - \lambda_2\psi_{ij}/\rho, a_{ij}^{k2} + \lambda_2\psi_{ij}/\rho\right) & \text{if } a_{ij}^{k1} > a_{ij}^{k2} + 2\lambda_2\psi_{ij}/\rho \\ \left(a_{ij}^{k1} + \lambda_2\psi_{ij}/\rho, a_{ij}^{k2} - \lambda_2\psi_{ij}/\rho\right) & \text{if } a_{ij}^{k2} > a_{ij}^{k1} + 2\lambda_2\psi_{ij}/\rho \\ \left(\frac{a_{ij}^{k1} + a_{ij}^{k2}}{2}, \frac{a_{ij}^{k1} + a_{ij}^{k2}}{2}\right) & \text{if } \left|\, a_{ij}^{k1} - a_{ij}^{k2}\right| \leq 2\lambda_2\psi_{ij}/\rho \end{cases}. \tag{13}$$

When $\lambda_1 > 0$, the solution to (12) can be obtained through soft-thresholding (13) by $\lambda_1\omega_{ij}$. Here the soft-thresholding is defined as $S(x, c) = \mathrm{sign}(x)\left(|x| - c\right)_+$, where $a_+ = \max(a, 0)$.

2.2.3 Complete ADMM algorithm

Based on the augmented Lagrangian, the complete ADMM algorithm for (4) is given in Algorithm 1. We find it is useful in practice to vary the value of penalty parameter ρ for each iteration. Therefore, we update ρ in each iteration based on the primal residues and dual residues. We present the update rule for ρ at the end of this section. Please note that according to the ADMM algorithm, the estimates $\hat{\Theta}^{k1}$ and $\hat{\Theta}^{k1}$ are not exactly sparse, while $\hat{\mathbf{Z}}^{k1}$ and $\hat{\mathbf{Z}}^{k1}$ are sparse which are obtained through soft-thresholding.

2.2.4 Stop criteria

Let Θ_t^{kc}, $\mathbf{Z}_t^{kc}$ and $\mathbf{U}_t^{kc}$ denote the estimates at the tth iteration. The primal residual [1] is defined as

$$\mathbf{P_t} = \begin{bmatrix}\Theta_t^{k1} \\ \Theta_t^{k2}\end{bmatrix} - \begin{bmatrix}\mathbf{Z}_t^{k1} \\ \mathbf{Z}_t^{k2}\end{bmatrix}, \tag{14}$$

Algorithm 1 ADMM algorithm for optimization problem (4)

- **Inputs:** Sample covariance matrices $\mathbf{S}^{k1}$ and $\mathbf{S}^{k2}$, sample size n_1 and n_2, penalty parameter $\rho = 1$, $\mu = 10$, $\tau^{\text{incr}} = 2$ and $\tau^{\text{decr}} = 2$
- **Output:** Estimated precision matrices, $\hat{\mathbf{\Theta}}^{k1}$ and $\hat{\mathbf{\Theta}}^{k1}$, and their sparse approximation, $\hat{\mathbf{Z}}^{k1}$ and $\hat{\mathbf{Z}}^{k1}$
- **Initialize:** $\mathbf{\Theta}^{kc} = \mathbf{I}$, $\mathbf{Z}^{kc} = \mathbf{I}$, $\mathbf{U}^{kc} = \mathbf{0}$ for $c = 1, 2$
- **while** *Not converged* **do**
  1. $\mathbf{\Theta}^{k1} \leftarrow \text{Expand}\left(\mathbf{Z}^{k1} - \frac{1}{\rho}\left(n_1\mathbf{S}^{k1} + \mathbf{U}^{k1}\right), \rho,\ n_1\right)$;
  2. $\mathbf{\Theta}^{k2} \leftarrow \text{Expand}\left(\mathbf{Z}^{k2} - \frac{1}{\rho}\left(n_2\mathbf{S}^{k2} + \mathbf{U}^{k2}\right), \rho,\ n_2\right)$;
  3. Update $\mathbf{Z}^{k1}$ and $\mathbf{Z}^{k2}$ through solving problem (11);
  4. $\mathbf{U}^{k1} \leftarrow \mathbf{U}^{k1} + \rho * \left(\mathbf{\Theta}^{k1} - \mathbf{Z}^{k1}\right)$;
  5. $\mathbf{U}^{k2} \leftarrow \mathbf{U}^{k2} + \rho * \left(\mathbf{\Theta}^{k2} - \mathbf{Z}^{k2}\right)$;
  6. Update ρ according to (18).
- Output $\mathbf{\Theta}^{k1}$, $\mathbf{\Theta}^{k1}$, $\mathbf{Z}^{k1}$ and $\mathbf{Z}^{k1}$

---

and the dual residual [1] is defined as

$$\mathbf{D_t} = \rho\left(\begin{bmatrix}\mathbf{Z}_t^{k1}\\ \mathbf{Z}_t^{k2}\end{bmatrix} - \begin{bmatrix}\mathbf{Z}_{t-1}^{k1}\\ \mathbf{Z}_{t-1}^{k2}\end{bmatrix}\right). \tag{15}$$

We consider the process converges if both primal residual and dual residual are sufficiently small [1]. More specially, we introduce small positive constants ϵ^{abs} and ϵ^{rel}, and declare $\mathbf{P_t}$ and $\mathbf{D_t}$ small if

$$\|\mathbf{P_t}\|_F \le \sqrt{2}p\epsilon^{\text{abs}} + \epsilon^{\text{rel}} \max\left\{\left\|\begin{bmatrix}\mathbf{\Theta}_t^{k1}\\ \mathbf{\Theta}_t^{k2}\end{bmatrix}\right\|_F, \left\|\begin{bmatrix}\mathbf{Z}_t^{k1}\\ \mathbf{Z}_t^{k2}\end{bmatrix}\right\|_F\right\}, \tag{16}$$

and

$$\|\mathbf{D_t}\|_F \le \sqrt{2}p\epsilon^{\text{abs}} + \epsilon^{\text{rel}} \left\|\begin{bmatrix}\mathbf{U}_t^{k1}\\ \mathbf{U}_t^{k2}\end{bmatrix}\right\|_F. \tag{17}$$

We set $\epsilon^{\text{abs}} = 10^{-3}$ and $\epsilon^{\text{rel}} = 10^{-3}$ as suggested by Boyd *et al.* [1]. The choice of the value of ϵ^{abs} depends on the scale of the variable values.

2.2.5 Varying penalty parameter

In practice, it is useful to use different penalty parameter ρ for each iteration, which might improve the convergence as well as make performance less dependent on the initial value of penalty parameter [1]. Therefore, we update the value of ρ in the tth iteration according to the primal residuals and dual residuals:

$$\rho \leftarrow \begin{cases} \tau^{\text{incr}}\rho & \text{if } \|\mathbf{P_t}\|_F > \mu\,\|\mathbf{D_t}\|_F \\ \rho/\tau^{\text{decr}} & \text{if } \|\mathbf{D_t}\|_F > \mu\,\|\mathbf{F_t}\|_F \\ \rho & \text{otherwise} \end{cases} \tag{18}$$

where $\mu > 1$, $\tau^{\text{incr}} > 1$ and $\tau^{\text{decr}} > 1$ are the adaptation parameters. We set $\mu = 10$, $\tau^{\text{incr}} = 2$ and $\tau^{\text{decr}} = 2$ according to [1].

2.3 Complete algorithm of the TDJGL model

By taking Equation (4) into Equation (3) in the main text, the TDJGL model can be written as follows:

$$\begin{aligned}\min_{\{\mathbf{\Theta}\}} \quad & \sum_{k=1}^{K}\sum_{c=1}^{2} n_c\left(\operatorname{tr}\left(\mathbf{S}^{kc}\mathbf{\Theta}^{kc}\right) - \log\det\left(\mathbf{\Theta}^{kc}\right)\right) + \lambda_1 \sum_{i\neq j}\sqrt{\sum_{k=1}^{K}\sum_{c=1}^{2}\left|\theta_{ij}^{kc}\right|} + \lambda_2 \sum_{i,j}\sqrt{\sum_{k=1}^{K}\left|\theta_{ij}^{k1} - \theta_{ij}^{k2}\right|} \\ \text{s.t.} \quad & \mathbf{\Theta}^{kc} \in S_{++}^{p},\ \text{for } k = 1, \ldots, K \text{ and } c = 1, 2.\end{aligned} \tag{19}$$

Given the sample covariance matrices $\left\{\mathbf{S}^{kc}\right\}_{k=1,\ldots,K}^{c=1,2}$ and the two parameters λ_1 and λ_2, we can find the estimates of precision matrices $\left\{\hat{\mathbf{\Theta}}^{kc}\right\}_{k=1,\ldots,K}^{c=1,2}$ and their sparse approximation $\left\{\hat{\mathbf{Z}}^{kc}\right\}_{k=1,\ldots,K}^{c=1,2}$ using Algorithm 2. Here we first decomposed (19) into K individual subproblems (4) using the method of local linear approximation (see the main text). Then we

iteratively solve each subproblems until convergence. Because the estimates $\left\{\hat{\boldsymbol{\Theta}}^{kc}\right\}_{k=1,\ldots,K}^{c=1,2}$ should be not accurately sparse when they are obtained by using the ADMM algorithm, we use $\left\{\hat{\mathbf{Z}}^{kc}\right\}_{k=1,\ldots,K}^{c=1,2}$ in the experiments, which are introduced as sparse approximation of $\left\{\hat{\boldsymbol{\Theta}}^{kc}\right\}_{k=1,\ldots,K}^{c=1,2}$ by the ADMM algorithm (please refer to Algorithm 1).

Algorithm 2 Complete algorithm of the TDJGL model (19)

- **Inputs:** The $2K$ sample covariance matrices $\left\{\mathbf{S}^{kc}\right\}_{k=1,\ldots,K}^{c=1,2}$, and the regularization parameters λ_1 and λ_2
- **Output:** The $2K$ estimated precision covariance matrices $\left\{\hat{\boldsymbol{\Theta}}^{kc}\right\}_{k=1,\ldots,K}^{c=1,2}$ and their sparse approximation $\left\{\hat{\mathbf{Z}}^{kc}\right\}_{k=1,\ldots,K}^{c=1,2}$
- **Main algorithms:**
  1. Initialize $\hat{\boldsymbol{\Theta}}^{kc}$ for $k = 1, \ldots, K$ and $c = 1, 2$.
  2. Compute the weights $\omega_{ij} = \frac{1}{2\sqrt{\sum_{k=1}^{K}\sum_{c=1}^{2}|\hat{\theta}_{ij}^{kc}|}}$ and $\psi_{ij} = \frac{1}{2\sqrt{\sum_{k=1}^{K}|\hat{\theta}_{ij}^{k1}-\hat{\theta}_{ij}^{k2}|}}$ for $i, j = 1, \ldots, p$.
  3. Update $\hat{\boldsymbol{\Theta}}^{k1}$ and $\hat{\boldsymbol{\Theta}}^{k2}$ for all $k = 1, \ldots, K$ by solving problem (4) using Algorithm 1.
  4. Repeat Steps 2 and 3 until the convergence condition is achieved.
  5. Output $\left\{\hat{\boldsymbol{\Theta}}^{kc}\right\}_{k=1,\ldots,K}^{c=1,2}$ and $\left\{\hat{\mathbf{Z}}^{kc}\right\}_{k=1,\ldots,K}^{c=1,2}$

2.4 Model selection

For the TDJGL model, parameter λ_1 controls the sparsity of the estimated networks, and parameter λ_2 has an influence on the sparsity of resulting differential networks. Therefore, the choice of λ_1 and λ_2 is critical. We determine the values of parameters in data-driven method via stability selection [6]. Stability selection, which seeks the parameters leading to most stable set of edges, has better results for network inference than other model selection methods including cross validation, Akaike information criterion and Bayesian information criterion [7, 8, 9, 10].

We choose λ_1 and λ_2 so as to use the least amount of regularization that simultaneously makes network sparse and stable. Here we resort to a recently developed stability selection method called StARS [7]. Because λ_1 mainly influences the sparsity and stability of resulting gene networks, while λ_2 mainly controls the sparsity and stability of estimated differential networks, we determine their values separately. We first determine the value of λ_1 while setting $\lambda_2 = 0$. Then we determine the value of λ_2 while setting λ_1 with the value chosen in the previous step.

We draw S random sample sets $\mathcal{D}_1, \ldots, \mathcal{D}_S$ from the $n = n_1 + n_2$ patients, each of size $0.8n$. For now, we choose λ_1 from a given vector of regularization parameter Λ_1 with setting $\lambda_2 = 0$. We estimate $2K$ networks $\left\{\hat{\mathbf{E}}_s^{kc}(\lambda_1, \lambda_2)\right\}_{k=1,\ldots,K}^{c=1,2}$ for each $\mathcal{D}_s$ and each λ_1 from Λ_1. The optimal value of λ_1 controls the average variance over the edges of the networks inferred from sub-sampled data:

$$\lambda_{1,\text{opt}}^{(\lambda_2)} = \arg\min_{\gamma\in\Lambda_1}\left\{\max_{\lambda_1\geq\gamma}\left(\sum_{k=1}^{K}\sum_{c=1}^{2}\left(\sum_{i<j}2\bar{a}_{ij}^{kc}(\lambda_1,\lambda_2)\left(1-\bar{a}_{ij}^{kc}(\lambda_1,\lambda_2)\right)\Big/\binom{p}{2}\right)\Big/2K\right)\leq\beta\right\}, \tag{20}$$

where $\bar{a}_{ij}^{kc}(\lambda_1, \lambda_2) = \frac{1}{S}\sum_{s=1}^{S}\mathcal{I}\left((i,j) \in \hat{\mathbf{E}}_s^{kc}(\lambda_1, \lambda_2)\right)$. Here we present StARS using a completeness form. Interested reader is referred to [7].

After determining λ_1, we choose λ_2 from a given vector of regularization parameter Λ_2 according to stability of inferred differential networks. For now, we set $\lambda_1 = \lambda_{1,\text{opt}}^{(\lambda_2)}$ which is determined above. We estimate $2K$ networks $\left\{\hat{\mathbf{E}}_s^{kc}(\lambda_1, \lambda_2)\right\}_{k=1,\ldots,K}^{c=1,2}$ for each $\mathcal{D}_s$ and each λ_2 from Λ_2. Then we construct K differential networks, $\left\{\widehat{\mathbf{DE}}_s^{k}(\lambda_1, \lambda_2)\right\}_{k=1,\ldots,K}$, based on the estimated networks. The optimal value for λ_2 is chosen according to the average variance over the differential edges inferred from sub-sampled data:

$$\lambda_{2,\text{opt}}^{(\lambda_1)} = \arg\min_{\gamma\in\Lambda_2}\left\{\max_{\lambda_2\geq\gamma}\left(\sum_{k=1}^{K}\left(\sum_{i<j}2\bar{b}_{ij}^{k}(\lambda_1,\lambda_2)\left(1-\bar{b}_{ij}^{k}(\lambda_1,\lambda_2)\right)\Big/\binom{p}{2}\right)\Big/K\right)\leq\beta\right\}, \tag{21}$$

where $\bar{b}_{ij}^{k}(\lambda_1, \lambda_2) = \frac{1}{S}\sum_{s=1}^{S}\mathcal{I}\left((i,j) \in \widehat{\mathbf{DE}}_s^{kc}(\lambda_1, \lambda_2)\right)$.

In this study, we set the number of random sample sets $N = 20$ and the stability parameter $\beta = 0.01$.

2.5 Criteria for platinum response groups

The criterion that has been used in [11, 12] is adopted to define platinum-based chemotherapy response groups. In particular, we download the clinical information (Biotab format) of ovarian tumors from the TCGA website. We obtain the drug information from the ''nationwidechildrens.org_clinical_drug_ov.txt'' file. Here we only consider platinum-based drugs (carboplatin, cisplatin, carbo) with regimen indication ''ADJUVANT'' and ''OTHER, SPECIFY IN NOTES''. The cancer progression information are obtained from the ''nationwidechildrens.org_clinical_follow_up_v1.0_nte_ov.txt'' file. New tumors with ''new_tumor_event_dx_evidence = [Not Available]'' and ''new_neoplasm_event_type = [Unknown]'' are not considered. The follow-up information of tumors with no progression are obtained from the ''nationwidechildrens.org_clinical_follow_up_v1.0_ov.txt'' file. For tumors with progression, it is defined as platinum-resistant if the new tumor occurs within 6 months of the end of primary treatment ($days\_to\_new\_tumor\_event\_after\_initial\_treatment - days\_to\_drug\_therapy\_end \leq 180\ days$), and it is defined as platinum-sensitive otherwise ($days\_to\_new\_tumor\_event\_after\_initial\_t$[illegible] $days\_to\_drug\_therapy\_end > 180\ days$). For tumors with no progression, it is defined as platinum-sensitive if the follow-up interval is at least 6 months from the date of last primary treatment ($days\_to\_last\_followup - days\_to\_drug\_therapy\_end > 180\ days$). Among the 514 tumors that have all the three types of gene expression profiles, 340 tumors that have explicit cisplatinum status, with 242 platinum-sensitive tumors and 98 platinum-resistant tumors. The sensitive and resistant information for each sample is presented in Supplementary information 2.

2.6 Comparison with other graphical lasso models on the ovarian cancer data

We compare TDJGL with FGL, GGL and GL on the ovarian cancer data. For FGL, we run it separately for each platform and each time it is applied across the two patient groups. For GGL, we run it separately for each patient group and each time it is applied across all the three platforms. For GL, we run it separately for each patient group and each platform type. In order to provide interpretable results, we select the tuning parameters of the compared methods to give the similar number of edges and differential edges as those of TDJGL. Unlike TDJGL and FGL, GGL and GL cannot control the similarities of precision matrices between different patient groups. They tend to identify too much differential edges. To better interpret the results of GGL and GL, we sort the absolute values of differential scores in decreasing order, and take the top #DE (the number of differential edges identified by TDJGL) edges for each model.

A common challenge in evaluating gene network inference and differential network analysis using real data is the lack of the gold standards. That is, in our ovarian cancer data analysis, we cannot obtain the true gene networks in the platinum-resistant tumors and the platinum-sensitive tumors. Therefore, it is difficult to compare different methods in terms of the accuracy of identifying group-specific gene networks and differential networks. In this study, we adopt an alternative way to evaluate performance. First, we compare the methods based on the overlaps between edges inferred from different platforms, which can assess the consistency. A method that produces a greater number of edges shared by different platforms is more consistent. Then, we compare the hub nodes in the differential networks in terms of known drug resistance-related genes and cancer-related genes. A method that works better in capturing known functionally important genes in the differential networks might have better performance in inferring the differential networks.

We observe that overlaps between edges (and differential edges) identified by FGL and GL from the different platforms are quite low (Figures S7 and S9). For GGL which encourages a similar network structure across all platforms, more than half of identified edges are shared by all the three platforms (Figure S8 (a)-(b)). Because GGL does not consider the similarity of differential networks across platforms, a great number of differential edges detected by GGL are supported by only one platform (Figure S8 (c)). As mentioned in the main text, both gene networks and differential networks inferred by TDJGL share a great number of edges across all the three platforms (Figure S6). We also compare the hub nodes in the differential networks inferred by different methods. For FGL, GGL and GL, we consider the 18 genes which have the largest degree of connectivity as hub genes. From Table S1, we find that the set of hub genes determined by TDGJL includes more cisplatin resistance-related genes, drug resistance-related genes and cancer-related genes than those determined by the other three methods.

Table S1: The number of hub genes that have been reported as platinum resistance-related genes, drug resistance-related genes and cancer-related genes

Methods	$GEAR_{cisplatin}$	$GEAR_{drug}$	CGC
TDJGL	5	10	8
FGL	0	3	6
GGL	1	5	6
GL	1	3	4

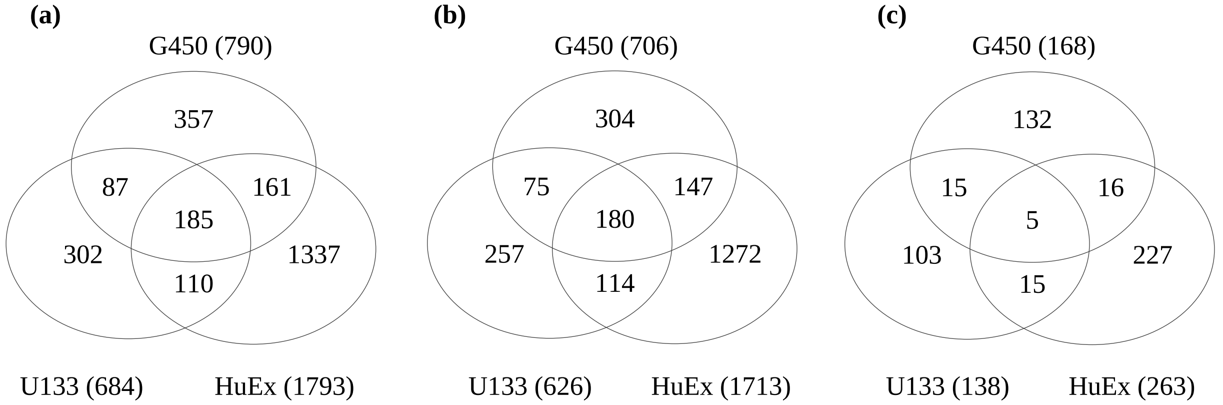

Figure S7: Overlaps between the edges (and differential edges) detected by FGL from the three platforms for (a) platinum-resistant tumors, (b) platinum-sensitive tumors and (c) differential networks.

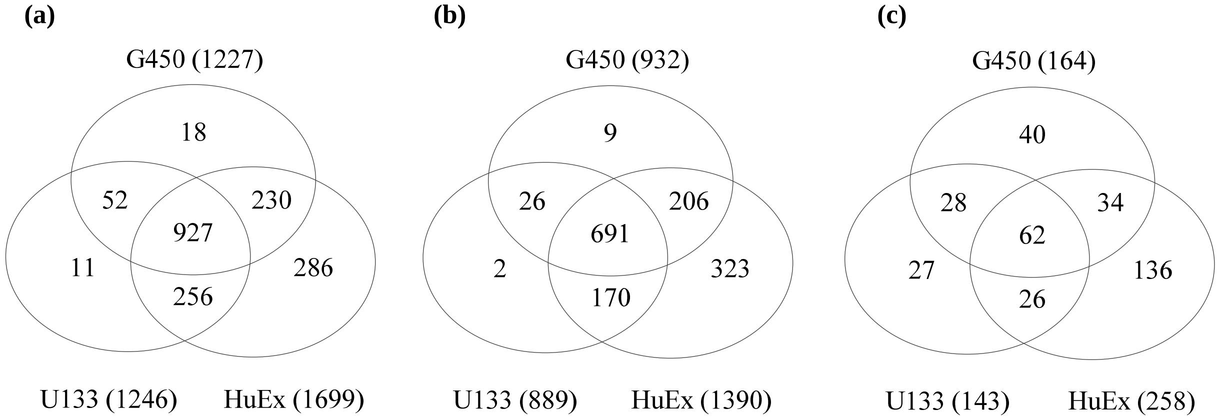

Figure S8: Overlaps between the edges (and differential edges) detected by GGL from the three platforms for (a) platinum-resistant tumors, (b) platinum-sensitive tumors and (c) differential networks.

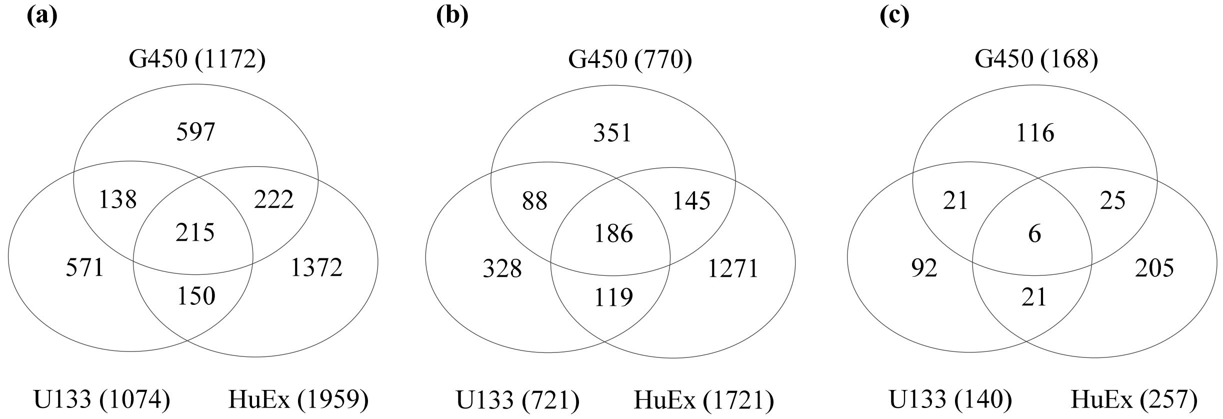

Figure S9: Overlaps between the edges (and differential edges) detected by GL from the three platforms for (a) platinum resistant tumors, (b) platinum-sensitive tumors and (c) differential networks.

References

[1] Stephen Boyd, Neal Parikh, Eric Chu, Borja Peleato, and Jonathan Eckstein. Distributed optimization and statistical learning via the alternating direction method of multipliers. *Foundations and Trends® in Machine Learning*, 3(1):1–122, 2011.

[2] Karthik Mohan, Palma London, Maryam Fazel, Daniela Witten, and Su-In Lee. Node-based learning of multiple gaussian graphical models. *The Journal of Machine Learning Research*, 15(1):445–488, 2014.

[3] Patrick Danaher, Pei Wang, and Daniela M Witten. The joint graphical lasso for inverse covariance estimation across multiple classes. *Journal of the Royal Statistical Society: Series B (Statistical Methodology)*, 76(2):373–397, 2014.

[4] Daniela M Witten and Robert Tibshirani. Covariance-regularized regression and classification for high dimensional problems. *Journal of the Royal Statistical Society: Series B (Statistical Methodology)*, 71(3):615–636, 2009.

[5] Holger Hoefling. A path algorithm for the fused lasso signal approximator. *Journal of Computational and Graphical Statistics*, 19(4):984–1006, 2010.

[6] Nicolai Meinshausen and Peter Bühlmann. Stability selection. *Journal of the Royal Statistical Society: Series B (Statistical Methodology)*, 72(4):417–473, 2010.

[7] Han Liu, Kathryn Roeder, and Larry Wasserman. Stability approach to regularization selection (stars) for high dimensional graphical models. In *Advances in neural information processing systems*, pages 1432–1440, 2010.

[8] Genevera I Allen and Zhandong Liu. A local poisson graphical model for inferring networks from sequencing data. *IEEE Transactions on NanoBioscience*, 12(3):189–198, 2013.

[9] Marinka Žitnik and Blaž Zupan. Gene network inference by fusing data from diverse distributions. *Bioinformatics*, 31(12):i230–i239, 2015.

[10] Zachary D Kurtz, Christian L Müller, Emily R Miraldi, Dan R Littman, Martin J Blaser, and Richard A Bonneau. Sparse and compositionally robust inference of microbial ecological networks. *PLOS Computational Biology*, 11(5):e1004226, 2015.

[11] . The Cancer Genome Atlas Research Network. Integrated genomic analyses of ovarian carcinoma. *Nature*, 474(7353):609–615, 2011.

[12] Sheida Nabavi, Daniel Schmolze, Mayinuer Maitituoheti, Sadhika Malladi, and Andrew H Beck. Emdomics: a robust and powerful method for the identification of genes differentially expressed between heterogeneous classes. *Bioinformatics*, page btv634, 2015.
